# Electrochemical properties of the TiO_2_(B) powders ball mill treated for lithium-ion battery application

**DOI:** 10.1186/1752-153X-7-174

**Published:** 2013-11-06

**Authors:** Bo-Ra Kim, Kang-Seop Yun, Hee-June Jung, Seung-Taek Myung, Sang-Chul Jung, Wooseung Kang, Sun-Jae Kim

**Affiliations:** 1 Institute/Faculty of Nanotechnology and Advanced Materials Engineering, Sejong University, Seoul 143-747, Korea; 2 Department of Environmental Engineering, Sunchon National University, Suncheon, Jeonnam 540-742, Korea; 3 Department of Metallurgical & Materials Engineering, Inha Technical College, Incheon 402-752, Korea

**Keywords:** TiO_2_(B), Anode materials, Ball-milling, Lithium-ion battery, Hydrothermal method

## Abstract

**Background:**

Belt or wire shaped TiO_2_(B) particles were synthesized for lithium ion battery application by a hydrothermal and heat treatment process. In order to facilitate TiO_2_(B)/C composites fabrication, the synthesized TiO_2_(B) particles were crushed into smaller sizes by ball milling.

**Results:**

Ball mill treated TiO_2_(B) particles of less than 1.0 μm with a fraction of anatase phase, compared to as-synthesized TiO_2_(B) particles with about 24 μm in average particle size, showed a significant improvement in the electrochemical properties. They showed a much improved stability in the charge–discharge cycles and irreversibility. They maintained about 98% of the initial capacity during 50 cycles while as-synthesized sample before ball mill treatment showed a gradual decrease in the capacity with the cycles. The irreversibility of 12.4% of as-synthesized sample was also greatly improved to 7% after ball milling treatment.

**Conclusions:**

Our results indicate ball mill treatment can be an economical way to improve electrochemical properties of TiO_2_(B) anode materials for lithium ion battery application.

## Introduction

Graphite has been extensively used as a negative electrode material due to its relatively high specific capacity and electrochemical stability [1-3]. However, several issues were brought about due to inherent electrochemical characteristics of graphite; electrodeposition of lithium at the surface of graphite during quick charging, and a solid electrolyte interface (SEI) layer formation by the secondary reactions between graphite and electrolyte. These problems deteriorated the performance of the graphite electrode, resulting in shorter lifetime and reduced capacity. Thus, an alternative electrode material has been actively explored to replace it. In particular, electric vehicles requiring fast charging need electrode materials not only experiencing little lattice distortions during charge–discharge cycles, but having a long lifetime, high stability, and high discharging capacity, etc. And most of all, they need to be fabricated at a reasonably low cost.

Among the promising materials to replace the graphite electrode, nano-structured TiO_2_ polymorphs have attracted much attention due to their high capacity and micro-channeled structure appropriate for intercalation and deintercalation of the Li ions during charge–discharge cycles [4,5]. The space existing between layers in the crystalline structure as well as within the TiO_2_ particles could facilitate the intercalation and deintercalation of Li^+^ ions during the process. Especially, TiO_2_(B) having relatively open structures compared to Rutile, Anatase, and Brookite has an obvious advantage for the intercalation and deintercalation of Li^+^ ions without severe lattice distortions [6,7]. The favorable structural characteristics of the TiO_2_(B) mentioned above are believed to improve the lifetime and performance of the battery [8-10]. In addition, the fabrication of TiO_2_(B) particles is relatively simple and easy to scale up for high volume production.

In this study, we obtained belt or wire shaped TiO_2_(B) particles to use as a negative electrode by a hydrothermal and heat treatment process. Ball milling process was utilized to crush the synthesized TiO_2_(B) particles into smaller sizes to facilitate TiO_2_(B)/C composites fabrication. The effects of ball mill treatment on the electrochemical performance of the TiO_2_(B) particles were evaluated in terms of microstructure and phase changes.

## Experimental details

For preparing TiO_2_(B) powder, 0.1 M P25 powder was at first put into 10 M aqueous NaOH solution, and then the solution was hydrothermally heated at 180°C for 24 hrs. The obtained particles were calcined at 450°C for 6 hrs in air, resulted in having TiO_2_(B) phase with nanobelt of thin and long length. After that ball-milling was conducted on TiO_2_(B), materials and electrochemical characterizations were carried out using SEM (Scanning Electron Microscopy, S-4700, Hitachi), XRD (X-Ray Diffraction, D/MAX 2500, Rigaku) and Raman spectroscopy (Renishow, invia Raman Microscope). As the milling time increased, particle size of TiO_2_(B) powders decreased from 24 μm to 0.2 μm through a 300 rpm, 3 hrs ball-milling. Here, the size was measured using PSA (Particle Size Analyzer with laser scattering, Photal ELS-800, Otsuka electronics).

Electrochemical properties were measured with R2032 coin-type cells. A metal lithium foil was used as the anode. Composite electrodes were prepared by mixing 80 wt% TiO_2_(B) powders, 10 wt% conductive Super-P, 10 wt% SBR-CMC binder, and N-methylpyrrolidone (NMP) solvent to form uniform slurry. A solution of 1 M LiPF6 dissolved in a mixture of ethylene carbonate (EC) and dimethyl carbonate (DMC) (1:1 w/w) was used as the electrolyte. The cells were charged and discharged between 1.0 and 3.0 V by applying a constant current of 50 mAg^-1^.

## Results and discussion

**Figure 1:**
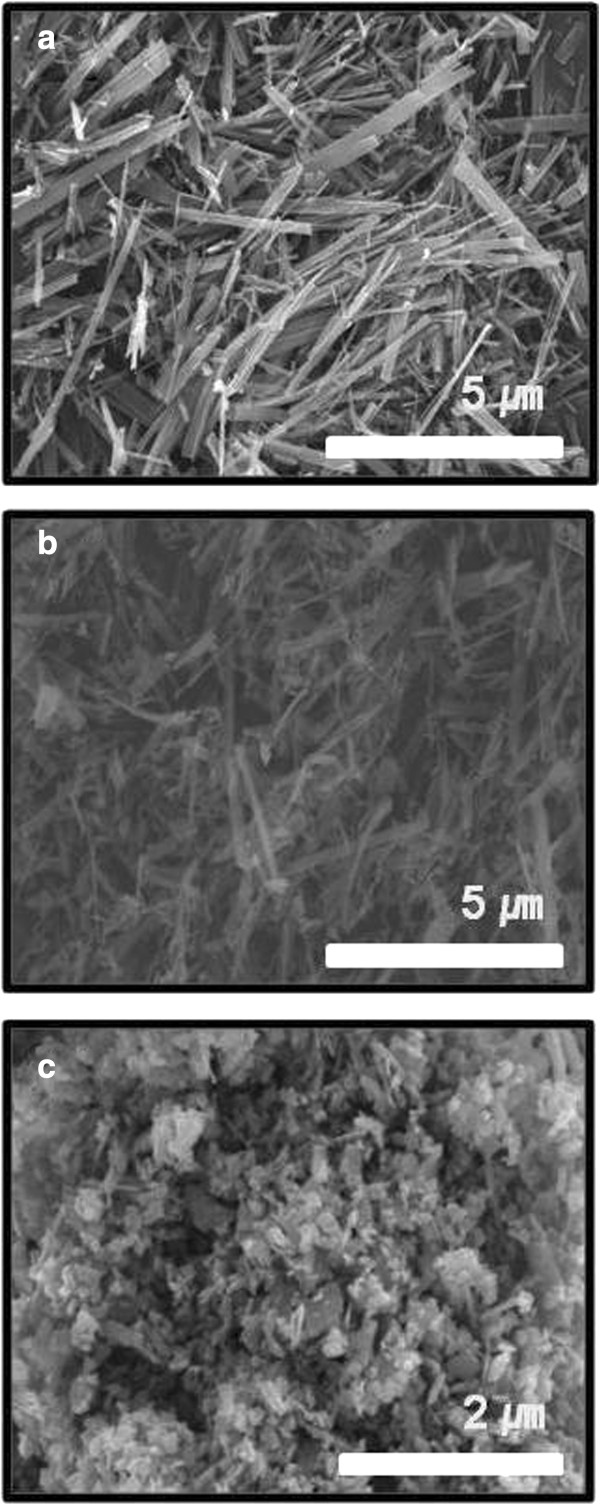
**SEM images for planetary-milled TiO**_**2**_**(B) powders with 300 rpm and various milling times. (a)** as-prepared TiO_2_(B), **(b)** 1 hr ball-milled TiO_2_(B), and **(c)** 3 hrs ball-milled TiO_2_(B).

**Figure 2:**
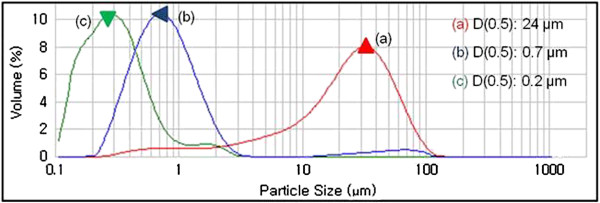
**Particle size distribution of planetary-milled TiO**_**2**_**(B) with milling times by laser scattering (PSA) shown in Figure **1**: (a)** as-prepared TiO_2_(B), **(b)** 1 hr ball-milled TiO_2_(B), and **(c)** 3 hrs ball-milled TiO_2_(B).

TiO_2_(B) powders were synthesized by the heat treatment of the rod or belt-shaped H-titanate powders at 450°C for 6 hrs, which had been prepared by a hydrothermal process. The combined process of the hydrothermal and heat treatment has been used for the synthesis of TiO_2_(B) powders [11,12]. The synthesized TiO_2_(B) powders were ball milled using a planetary type ball mill at a fixed rotation speed of 300 rpm for up to 3 hrs to make them smaller sizes. The changes of morphology and size of the powders with the milling time were observed under SEM as shown in Figure 1. The as-synthesized TiO_2_(B) powders were observed from Figure 1(a) to be long rod or belt shaped. The powders were broken into smaller sizes after 1 hr ball milling (Figure 1(b)). However, they became severely agglomerated after ball milling for 3 hrs as can be seen in Figure 1(c). The size distribution of the TiO_2_(B) powders with the milling time was graphed in Figure 2. As-synthesized TiO_2_(B) powders with an average of 24 μm became broken into smaller sizes of 0.7 and 0.2 μm after ball milling for 1 and 3 hrs, respectively.

**Figure 3:**
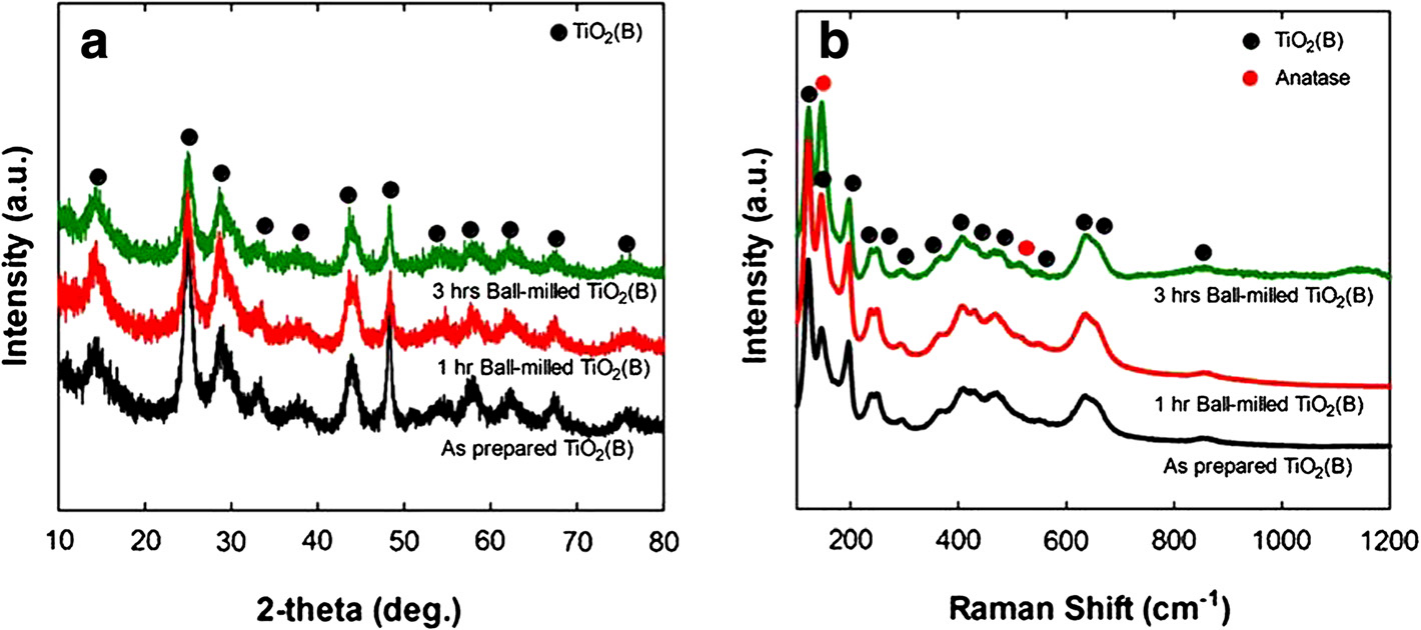
**XRD patterns (a) and Raman spectra (b) of the planetary ball-milled TiO**_**2**_**(B) samples used in Figure**1**.**

Figure 3 shows the XRD patterns and Raman spectra for the samples of as-synthesized and ball milled for up to 3 hrs. Typical peaks for TiO_2_(B) phase were observed from all the samples as in Figure 3(a). It was also noticed that the intensity of the main peak at 24.979 deg. was gradually decreased but the FWHM was increased with the milling time, indicating the particles were becoming smaller sizes. These results were found to be consistent with the SEM observations in Figure 1 for the size changes of the particles with the milling time.

Raman spectra showed that as-synthesized sample was composed of primarily TiO_2_(B) with the minor phase of anatase as seen in Figure 3(b). However, intensity of the anatase became stronger with the ball milling time, resulting in a dominant phase after ball milling for 3 hrs.

**Figure 4:**
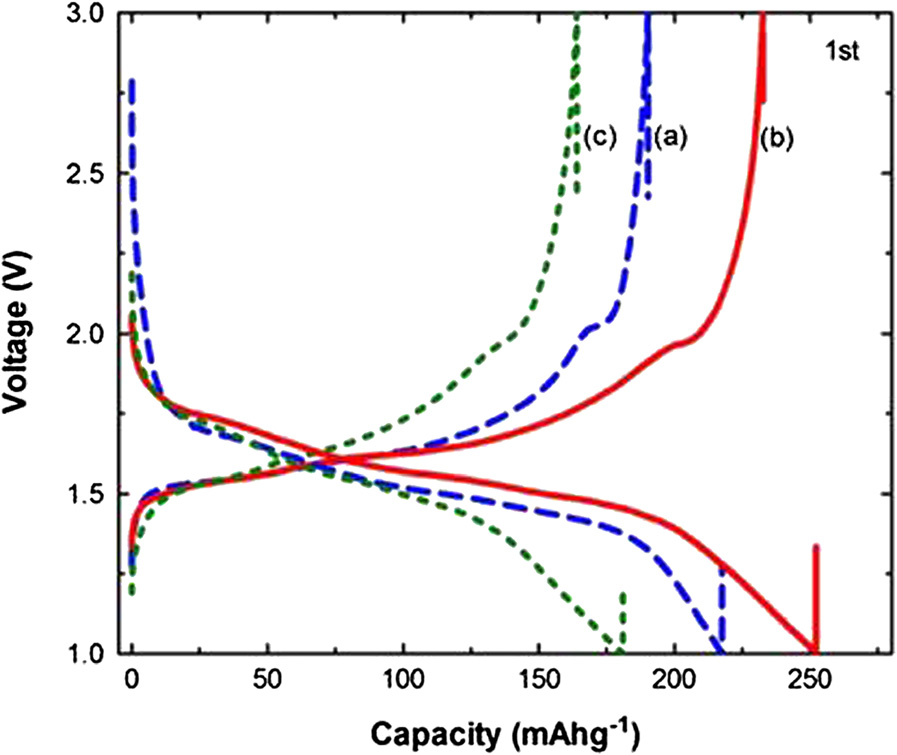
**The initial charge–discharge curves of planetary-milled TiO**_**2**_**(B) with milling times by laser scattering (PSA) shown in Figure **1**. (a)** as-prepared TiO_2_(B), **(b)** 1 hr ball-milled TiO_2_(B), and **(c)** 3 hrs ball-milled TiO_2_(B) at 0.2 C.

Figure 4 shows the initial charge–discharge performance for the TiO_2_(B) samples measured in the range of 1.0 – 3.0 V with a lithium foil as a counter electrode. As-synthesized TiO_2_(B) sample had the charging capacity of about 217 mAh/g, discharging capacity of 190 mAh/g, and the resultant irreversibility of about 12.4%. Meanwhile, the sample ball milled for 3 hrs was measured with the charging capacity of about 180 mAh/g, discharging capacity of 164 mAh/g.

The reduced charge–discharge capacity of the 3 hrs ball milled sample seemed to be caused by the presence of the strong anatase peaks identified with Raman spectra in Figure 2. Similar to our results, TiO_2_ anode materials with anatase or rutile phase were reported to have a relatively low charging capacity of about 150 – 200 mAh/g [13,14]. It is also well known that charge–discharge capacity is much affected by the particle size of the electrode materials; smaller particles show better performance [15,16]. On the other hand, TiO_2_(B) sample ball milled for 1 hr was measured to have a high charging capacity of 250 mAh/g and discharging capacity of 232 mAh/g, and irreversibility of 7%. Based on the results with 1 hr and 3 hrs ball milled samples, the performance was found to be affected positively by the smaller scale of particles, but negatively by the presence of anatase phase; the presence of anatase phase with a strong intensity in the 3 hrs ball milled sample even with smallest particles deteriorated the performance.

**Figure 5:**
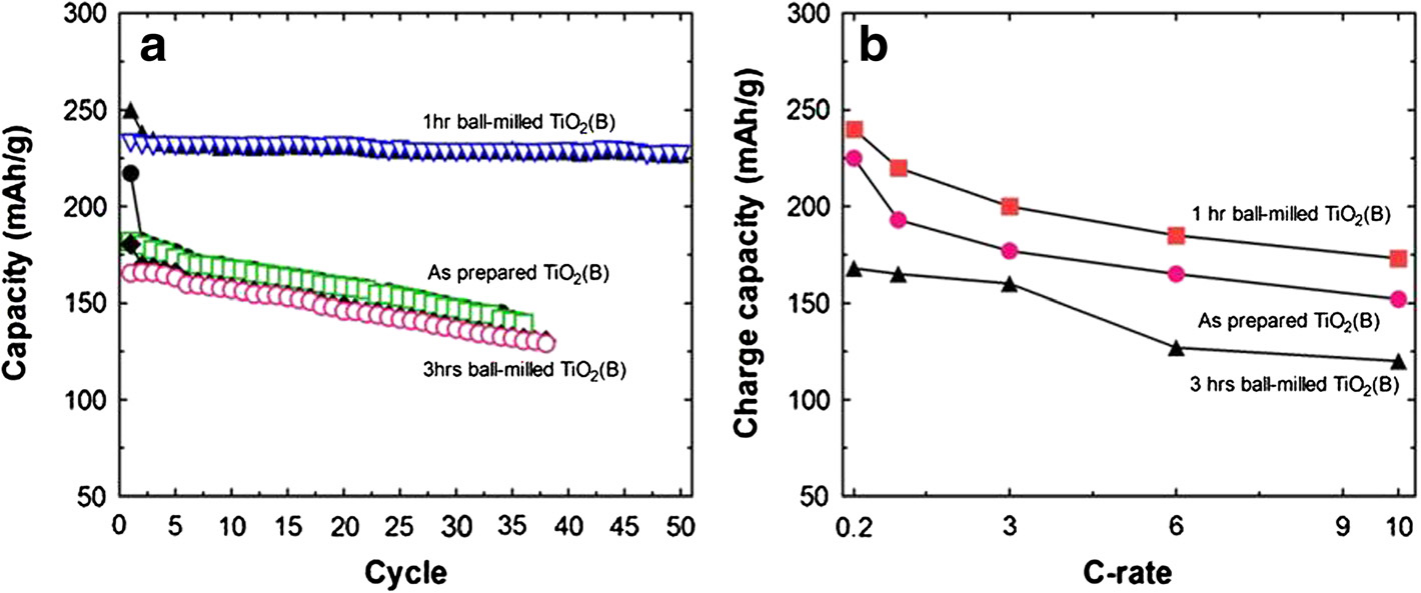
**Charge–discharge cycle (a) and C-rate characteristics (b) of planetary-milled TiO**_**2**_**(B) with milling times shown in Figure **1**.**

Cyclic stability of charge–discharge capacity and capacity variation as a function of C-rate was compared among the samples in Figure 5. As can be seen in Figure 5(a), TiO_2_(B) sample ball milled 1 hr showed a high degree of cyclic stability. About 98% of the initial discharge capacity was maintained even after 50 cycles; initial capacity of 232 mAh/g was reduced only to 227 mAh/g. On the other hand, as-synthesized sample and 3 hrs ball milled sample showed a gradual decrease of the capacity with the cycle suggesting smaller particle size without anatase phase in TiO_2_(B) was an optimum condition for the high electrochemical performance. Discharging capacity was gradually decreased with the C-rate regardless of the sample types as compared in graph Figure 5(b). But the gaps in the initial capacity among the samples were observed to be kept during the whole measurements in the C-rate range of 0.2 – 10 C.

## Conclusions

TiO_2_(B) particles were synthesized for lithium ion battery application by a hydrothermal and subsequent heat treatment processes. They were rod or thin belt shaped of about 24 microns in average size. For the efficient fabrication of TiO_2_(B)/C composite anode materials, the TiO_2_(B) particles were crushed into smaller sizes by ball milling process. Prolonged ball milling for up to 3 hrs produced particles with the average size of less than 1.0 μm. However, 3 hrs ball milled sample showed the anatase phase with a strong intensity by Raman spectra observation. Similar to other research results, the sample with the strong anatase peaks was measured to have a relatively low reversible capacity of 165 mAh/g compared to 190 mAh/g of as-synthesized sample. On the other hand, the sample ball milled for 1 hr, consisting of less than 1.0 μm particles with weak anatase peaks, showed much improved reversible capacity of 232 mAh/g.

TiO_2_(B) sample ball milled for 1 hr also showed a very high stability during charge–discharge cyclic tests. : It maintained about 98% of the initial discharge capacity during 50 cycles while as-synthesized and 3 hrs ball milled samples showed a gradual decrease in the capacity with the cycles.

Our results suggest that the electrochemical performance of TiO_2_(B) as a negative electrode material can be significantly improved with a simple and economical way of ball milling treatment.

## Competing interests

The authors declare that they have no competing interests.

## Authors’ contribution

BRK has carried out the experiments for the study, collected the data and analyzed them and has written the manuscript. KSY and HJJ have been involved by contributing their intellectual content for the research work. STM and SCJ have made their intellectual contributions in revising the manuscripts with their knowledgeable suggestions. WK and SJK were involved in interpretation of the data, drafting the manuscript, analyzing the results and critically revising it for intellectual content. The final manuscript has been read and approved by all the authors.
